# Partially Different Mechanisms of Social and Nonsocial Attention: Evidence From Changes in Cueing Effects and Underlying Frontal Cortex Processing Over Time

**DOI:** 10.1111/psyp.70286

**Published:** 2026-03-25

**Authors:** Michael K. Yeung, Yvonne M. Y. Han

**Affiliations:** ^1^ Department of Psychology The Education University of Hong Kong Hong Kong SAR China; ^2^ University Research Facility of Human Behavioral Neuroscience The Education University of Hong Kong Hong Kong SAR China; ^3^ Centre for Psychosocial Health The Education University of Hong Kong Hong Kong SAR China; ^4^ Department of Rehabilitation Sciences The Hong Kong Polytechnic University Hung Hom Hong Kong SAR China; ^5^ University Research Facility in Behavioral and Systems Neuroscience The Hong Kong Polytechnic University Hung Hom Hong Kong SAR China

**Keywords:** alerting, fNIRS, gaze, orienting, prefrontal cortex, social attention

## Abstract

Gaze conveys important information about one's intentions and likely object of reference. Because processes of attention may change over time, for reasons including fatigue or experience, this study aimed to compare mechanisms of gaze and arrow cueing effects by measuring across sessions. On two separate occasions, 39 young adults underwent a cueing paradigm with valid or invalid gaze or arrow cues, as well as neutral cues. Activation in frontal cortex regions implicated in the dorsal and ventral attention networks was examined during task performance using functional near‐infrared spectroscopy. Behavioral results showed comparable orienting (valid vs. neutral) and reorienting (invalid vs. valid) responses following gaze and arrow cues, which did not significantly change over sessions. However, the gaze cue elicited a significantly greater alerting effect (i.e., more benefits from the presence of the cue on reaction time) than the arrow cue in Session 2. Parallel to these behavioral findings, neuroimaging results indicated robust (de‐)activation during orienting and reorienting. Aligning with the greater alerting effect, target detection elicited significantly greater activation in the left posterior dorsomedial frontal cortex following gaze cues as opposed to arrow cues in Session 2. Therefore, insofar as changes over time are concerned, our findings offer converging evidence that gaze and arrow cues follow partially different attentional and neural mechanisms.

## Impact Statement

By observing changes over time, this study was among the first to demonstrate that social and nonsocial attention, assessed through gaze and arrow cueing, involve partially distinct psychological and neural mechanisms. Our findings suggest that social attention may be unique, with important implications for understanding social attention deficits. Additionally, the results support using functional near‐infrared spectroscopy to further investigate the neural mechanisms underlying social attention.

## 1 Introduction

Gaze conveys rich information about a person's likely object of reference and intentions; humans have an innate tendency to spontaneously follow the gaze of others and infer mental states based on its direction (Baron‐Cohen et al. 1995; Frischen et al. 2007). Gaze‐related attentional processes can be probed using adaptations of Posner's cueing paradigm (Posner 1980). In a conventional paradigm, participants view a central directional gaze cue. After a short time (e.g., 300 ms), an object appears on one side that is the same as or different from the gaze direction; the subject must identify the object or its location as fast as possible. One common finding is that reaction time (RT) is faster when the object appears in the same (i.e., valid) direction than in the opposite (i.e., invalid) direction of the gaze cue (Friesen and Kingstone 1998; McKay et al. 2021). This effect can be observed even when using gaze cues that are not predictive of the object location, or when using impoverished schematic face drawings (e.g., black pupils on white sclera; Friesen and Kingstone 1998; McKay et al. 2021; Ristic et al. 2002). Some researchers have referred to this validity effect as reflective of the orienting process. However, significantly faster RT is sometimes (Friesen and Kingstone 1998), but not always (Greene et al. 2011), observed following a valid directional cue than a nondirectional cue, indicating a facilitative orienting response before expectation violation. Therefore, the validity effect may be interpreted as reflecting the reorienting process (Corbetta et al. 2008; Joseph et al. 2015).

There has been a tremendous effort to determine whether the mechanisms of gaze processing are unique (Chacón‐Candia et al. 2023; Tipples 2002). Due to the evolutionary significance of gaze (Emery 2000), it has been suggested and found to automatically capture attention (Friesen and Kingstone 1998). Nevertheless, symbolic directional cues, such as arrows, have also been found to elicit a validity effect even though they are spatially nonpredictive (Frischen et al. 2007; Tipples 2002). One recent meta‐analysis of Posner's cueing paradigm found a similar magnitude of this validity effect between gaze and arrow cues (Chacón‐Candia et al. 2023), suggesting that the mechanisms underlying gaze and arrow processing for reorienting are similar, at least quantitatively (but see Marotta et al. 2018).

Another approach to understanding gaze and arrow cueing effects is to examine the underlying neural processing. There are two prominent frontoparietal attention networks in the brain (Corbetta and Shulman 2002; Vossel et al. 2014). The dorsal attention network consists of the intraparietal sulcus and frontal eye fields and was originally proposed to mediate endogenous attention—the voluntary, top‐down deployment of attention (Corbetta and Shulman 2002). The ventral attention network is right‐lateralized and consists of the temporoparietal junction and inferior frontal gyrus. It was originally proposed to mediate exogenous attention—the orientation of attention in a stimulus‐driven manner (Corbetta and Shulman 2002). Despite the identification of two distinct attention systems, emerging research has revealed a close interaction between them, blurring their strict separation (Suo et al. 2021; Vossel et al. 2014). The right anterior insula and right posterior inferior frontal gyrus have been suggested to be the critical hubs where the two attention systems meet (Cazzoli et al. 2021; Suo et al. 2021).

Some functional magnetic resonance imaging (fMRI) studies have compared the neural mechanisms underlying gaze and arrow cueing effects. Those studies, comparing directional vs. nondirectional cues without distinguishing between valid and invalid cues, have reported mixed results. Specifically, Greene et al. (2011) reported greater activation in the inferior frontal gyrus, premotor cortex, parietal cortex, and other regions for gaze cues than for arrow cues. In contrast, Hietanen et al. (2006) found activation in the frontal and supplementary eye fields (BA 6) and other cortical regions for arrow cues but not for gaze cues, with significant differences between the two cues. In addition, a recent meta‐analysis reported activation in the superior frontal gyrus for both gaze and arrow cues, but the locus of activation was different for the two cue types (Salera et al. 2024). This meta‐analysis also identified greater activation in the parietal cortex triggered by arrow than gaze cues. Moreover, one fMRI study made a distinction among neutral, valid, and invalid trials (Joseph et al. 2015). The valid vs. neutral cue contrast showed that only the arrow cue induced a significant orienting response in the occipital cortex. In contrast, the invalid vs. valid cue contrast—which involves a violation of expectation—revealed a reorienting response involving the frontal eye fields and other cortical regions for the gaze cue, but not the arrow cue. Therefore, whether gaze and arrow cues engage different neural processing remains uncertain.

Most studies have investigated and compared gaze and arrow cueing effects using only single occasions. However, studies using the cueing paradigm have shown that certain attentional processes can change over time due to fatigue or experience (Bednarek et al. 2021; Ishigami and Klein 2010; Yeung 2023a). Studies using the Attention Network Test with nondirectional cues showed that the alerting function (i.e., RT facilitated by the presence of a cue) and orienting process were relatively robust across sessions (Ishigami and Klein 2010), although alerting efficiency might decline throughout a session due to fatigue (Yeung 2023a). In contrast, the time to reorient attention following a valid vs. invalid peripheral cue significantly shortened across sessions, indicating that voluntary control may gradually be employed to overcome the tendency to orient according to a spatially nonpredictive cue (Yeung 2023a). Currently, little is known about whether gaze and arrow cueing effects exhibit systematic changes over time, and if such changes occur, whether they are similar or different for the two cue types. Comparing changes over time with underlying neural processing can generate new perspectives that inform the shared and divergent mechanisms of social and nonsocial attention, with important implications for understanding populations with altered social information processing, such as autistic individuals (Yi et al. 2022).

In the present study, we used functional near‐infrared spectroscopy (fNIRS) to examine and compare changes in social and nonsocial cueing effects and underlying frontal cortex processing over time. Because fNIRS places little demand on participants and is quite resistant to ocular and motion artifacts (Ferrari and Quaresima 2012), this method is deemed appropriate for studying attention with the cueing paradigm, which often involves eye and head movements (Khan et al. 2009). Based on the existing literature (e.g., Friesen and Kingstone 1998; Greene et al. 2011), participants' RT was expected to be hindered by gaze and arrow invalid cues and possibly facilitated by valid cues. Due to accumulated knowledge about the cues, we expected that the gaze and arrow cueing effects would change over time. Due to the evolutionary significance of gaze leading to more attention allocated to relevant cues, we also hypothesized that these changes would be greater for gaze cues compared to arrow cues, implying different processing mechanisms.

## 2 Methods

### 2.1 Participants

Forty young adults aged 18–25 years were recruited via advertisements on the campus of The Hong Kong Polytechnic University. Exclusion criteria were based on self‐reports, including (1) a history of neurological or psychiatric disorder; (2) traumatic brain injury that required hospitalization; (3) currently taking psychotropic medication; and (4) left‐handedness as determined by the short form of the Edinburgh Handedness Inventory (Veale 2014). All participants self‐reported normal or corrected‐to‐normal vision. One participant was excluded due to a technical error leading to missing data in Session 1. Therefore, the analytic sample comprised 39 participants (21 males, 18 females) with a mean age of 21.6 years (SD = 1.3 years). Although effect sizes were incompletely reported in previous fMRI studies, a sensitivity analysis assuming *n* = 39, a power of 0.80, and a Bonferroni‐corrected alpha of 0.0083 revealed that the present study had sufficient power to detect a moderate effect size (Cohen's *f* = 0.295). Note that the current sample size was approximately double that of earlier fMRI studies that reported significant effects (Greene et al. 2011; Hietanen et al. 2006; Joseph et al. 2015). Written informed consent was obtained from each participant before the experiment. This study was approved by the Human Subjects Ethics sub‐committee at The Hong Kong Polytechnic University (HSEARS20210315010) and conducted in compliance with the Declaration of Helsinki.

### 2.2 Procedure

After screening for eligibility, individuals were invited to the University Research Facility in Behavioral and Systems Neuroscience at The Hong Kong Polytechnic University to participate in this study, which took place within an overarching project on frontal cortex processing during task performance across sessions (Yeung 2023b; Yeung and Han 2023). Each participant attended two sessions that were separated by approximately 3 weeks (*M* = 21.2 days, SD = 0.9 days). They were instructed to avoid consumption of caffeine and alcohol on the days of the study.

### 2.3 Cueing Task

**FIGURE 1:**
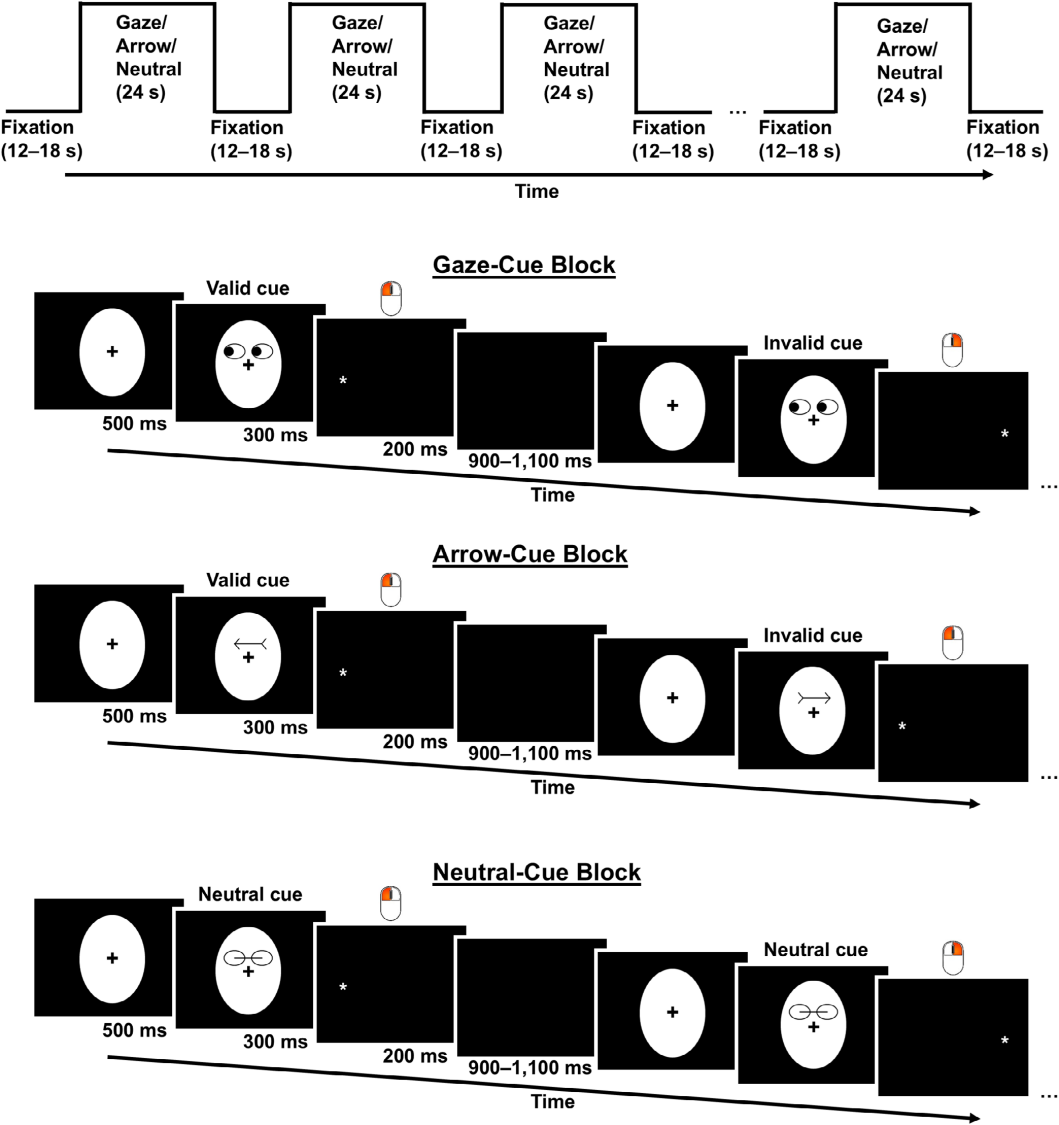
Flow of the cueing paradigm. The asterisk (*) indicates the target. The order of the three block conditions and the order of valid and invalid cues within the gaze‐cue and arrow‐cue blocks were randomized. These orders varied for each participant and session.

The cueing paradigm was adapted from fMRI studies (Hietanen et al. 2006; Joseph et al. 2015). The flow of the paradigm is presented in Figure 1. The task had a mixed design, with three block conditions: (1) gaze cue; (2) arrow cue; and (3) neutral cue. The order of these three block types was randomized, and this order was different across participants and sessions. Each condition had four blocks of 12 trials each, for a total of 12 blocks. Neither condition occurred more than twice in a row. A rest period of 12–18 s was inserted between two blocks to establish a baseline. For the gaze and arrow conditions, there were six valid and six invalid trials presented in a randomized order in each block. The order was different across individuals and sessions. Participants underwent this paradigm while activation in various frontal cortex ROIs was measured by fNIRS.

Each trial lasted an average of 2000 ms, beginning with a 500 ms fixation with an oval shape. A central cue predictive of the time of occurrence but not the location of the target then appeared for 300 ms. In the gaze condition, a pair of schematic eyes with a gaze to the left or right was shown. In the arrow condition, an arrow pointing to the left or right appeared. In the neutral condition, a nondirectional cue that mimicked the gaze and arrow cue was presented. Schematic eyes were used to match the arrow and neutral cues in visual complexity. The cues subtended 3.3° of visual angle.

After cue presentation, an asterisk target appeared for 200 ms on either the left or right side of the screen, located at 8.2° of visual angle from the center. Participants were asked to identify the target location by pressing the left or right button. A trial was considered valid if the target appeared in the same direction as the gaze or arrow cue and invalid if the target appeared opposite to the cued direction. All trials had a nondirectional cue in the neutral condition. The trial ended with a varying interstimulus interval of 900–1100 ms (*M* = 1000 ms).

Before the actual task commenced, participants sat 70 cm away from the screen and were informed of the task instructions. They were asked to sit still and minimize head movements throughout the task. Participants then practiced each task condition three times or until achieving 80% accuracy, whichever occurred first. Stimuli were presented on a 17‐in. Dell monitor with a 5:4 aspect ratio using E‐Prime 3.0 (Psychology Software Tools, Pittsburgh, PA, USA).

A mixed design was adopted because we originally planned to examine both sustained and transient cue effects. Because a schematic gaze and arrows matched in visual complexity were used, only one neutral condition with nondirectional cues was employed to account for the alerting effect offered by a temporally predictive cue. Because statistical analyses revealed no significant differences in the level of sustained activation among the three block types,^1^ we report the transient effects associated with gaze and arrow cues.

### 2.4 fNIRS Measurements

**FIGURE 2:**
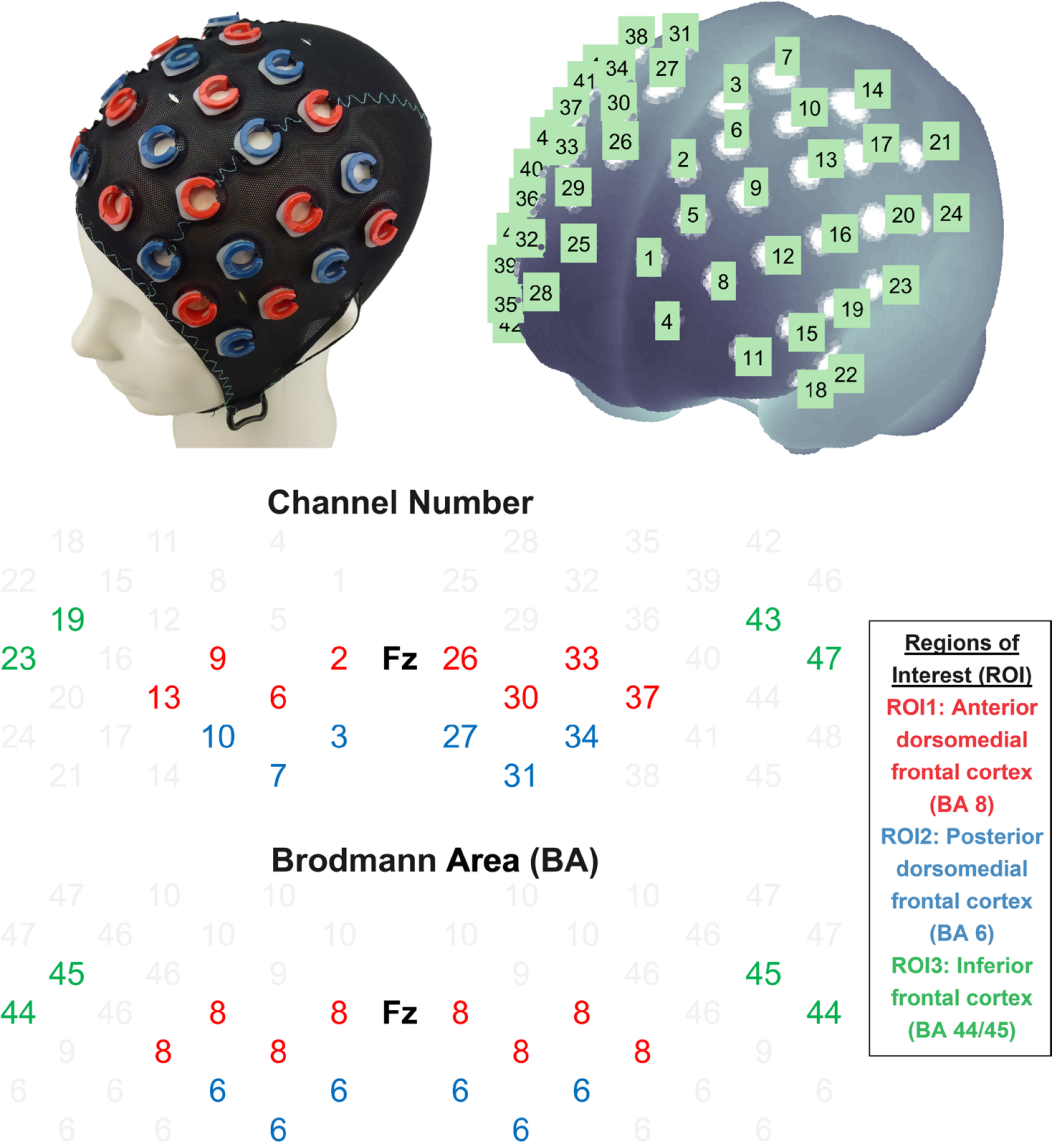
Functional near‐infrared spectroscopy setup and prefrontal cortex regions of interest.

The ETG‐4000 fNIRS device (Hitachi Medical Co., Tokyo, Japan) was used to measure hemodynamic changes in various frontal cortex regions during the cueing task. The setup is illustrated in Figure 2. The device used 695‐nm and 830‐nm lights and sampled data at 10 Hz. Participants wore an EasyCap that fit their head size, and the cap was mounted with 16 emitters and 16 detectors. The emitters and detectors were alternatingly positioned and arranged in two 4 × 4 arrays (i.e., equivalent to a 4 × 8 matrix), centering at Fz overall. Depending on the head size, the optodes were separated by 29–31 mm to achieve fixed locations with respect to the 10–20 positions. Based on the optodes' coordinates in the 10–20 system, the fNIRS probe and channel positions were rendered onto the Montreal Neurological Institute standard brain using the NFRI toolbox (Singh et al. 2005).

### 2.5 fNIRS Data Preprocessing

The HomER3 package and custom MATLAB scripts (version R2020a, MathWorks Inc., Natick, MA, USA) were used to preprocess the fNIRS data (Huppert et al. 2009). First, channels with overall mean raw intensities of > 4.9 units (maximum = 5.0 units; saturated channels) and overall signal‐to‐noise ratios of < 20 dB (noisy channels) were rejected (Yücel et al. 2021). On average, 5.2 and 5.0 out of 48 channels were rejected for the first and second sessions (SD = 5.3 and SD = 5.8, respectively). Negative intensity values were corrected by offsetting. The raw intensity signals were then converted to optical density changes.

The temporal derivative distribution repair algorithm was applied to remove baseline shift and spike artifacts (Fishburn et al. 2019). Systematic confounding was removed using principal component analysis. The first component associated with maximal correlation with the global average signal was removed for all participants (Carbonell et al. 2011). Next, a 0.01–0.5‐Hz bandpass filter was applied to remove cardiac artifacts, high‐frequency noise, and slow drifts. The filtered data were then converted to changes in HbO and HbR via the modified Beer–Lambert law. The differential pathlength factor was corrected for wavelength and age (Scholkmann and Wolf 2013). Because brain activation is associated with negatively correlated changes in HbO and HbR, the correlation‐based signal improvement (CBSI) method was used to combine the two chromophores (Cui et al. 2010). Statistical analyses were performed on the CBSI‐corrected HbO. For brevity, the change in CBSI‐corrected HbO will be referred to as the change in HbO.

A general linear model approach was adopted to estimate activation underlying orienting and reorienting. The canonical hemodynamic response function (HRF) was chosen to be the basis function (Ashburner et al. 2014). To isolate the orienting and reorienting processes, the canonical HRF was convolved with the delta function of the cue onset and target onset, respectively. The models were solved using the ordinary least squares method, yielding beta values. A higher beta value represents greater activation.

An ROI approach was adopted for subsequent analyses. Based on virtual registration and the Brodmann area atlas (Singh et al. 2005), six frontal cortex ROIs implicated in the dorsal and ventral attention networks were selected, including the bilateral anterior dorsomedial frontal cortex including the frontal eye fields (BA 8), posterior dorsomedial frontal cortex including the supplementary eye fields (BA 6), and inferior frontal cortex (BA 44/45) (Salera et al. 2024; Vossel et al. 2014). Beta values were averaged across channels, excluding rejected channels, for each ROI (Figure 2).

### 2.6 Data Analysis

Behavioral variables were accuracy and mean reaction time (RT). The mean RT was calculated after excluding incorrect trials and outlier RTs that were < 150 ms or > 2.5 SDs above the respective mean (Yeung et al. 2021). The mean total percentage of excluded incorrect trials and outlier RTs was 1.8% (SD = 7.3%) and 3.3% (SD = 1.8%) in Session 1, and 1.4% (SD = 3.3%) and 3.4% (SD = 1.7%) in Session 2. In addition, fNIRS variables included the beta values representing the change in HbO in the six frontal cortex ROIs. The neutral condition served as a common control for the gaze and arrow conditions. The orienting process was examined by comparing valid and neutral trials, and the reorienting response was investigated by comparing invalid and valid trials.

To test the orienting process, a linear mixed model with the subject as a random factor and session (first, second), cue (gaze, arrow), and trial type (valid, neutral) as fixed factors was conducted for each metric. To test the reorienting process, a similar linear mixed model was conducted for each metric, except with the trial types as valid and invalid trials. Degrees of freedom were estimated using Satterthwaite's formula, and the models were solved using the restricted maximum likelihood method. The alpha level was set at 0.05. For fNIRS, the *p*‐value threshold was Bonferroni‐corrected to 0.0083 (0.05/6) due to six frontal cortex ROIs. All statistical analyses were performed using IBM SPSS Statistics for Windows, version 26.0 (IBM Corp., Armonk, NY, USA).

## 3 Results

### 3.1 Behavioral Results

**FIGURE 3:**
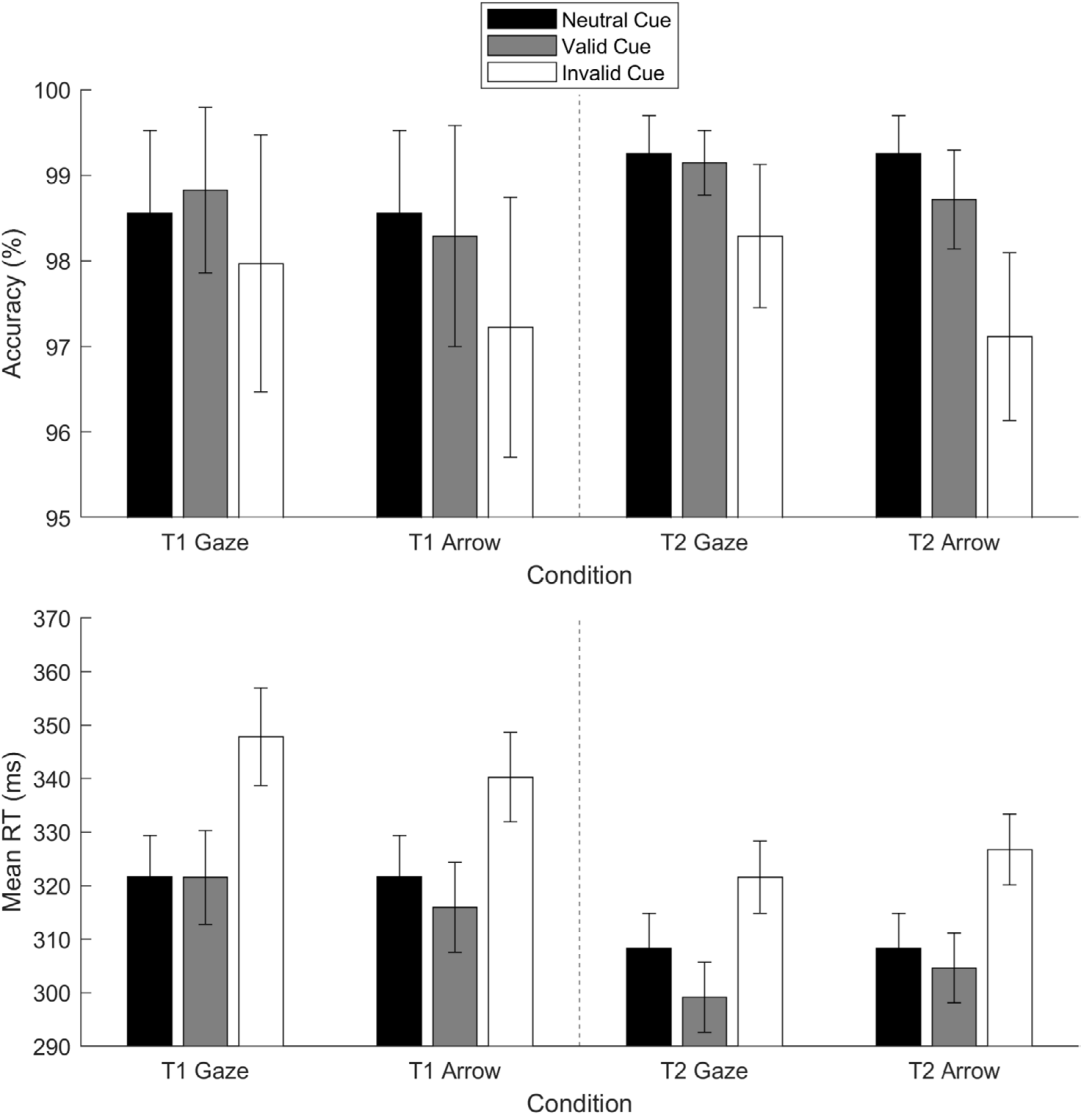
Accuracy and mean reaction time (RT) on the cueing tasks. T1, Time 1; T2, Time 2. Error bars indicate one standard error ± the mean.

The accuracy and mean RT during the cueing task are presented in Figure 3. The descriptive statistics of the corresponding orienting and reorienting metrics are shown in Table 1.

**TABLE 1:** Accuracies and mean reaction times representing the orienting (valid > neutral cue trials) and reorienting (invalid > valid cue trials) processes.

Process	Session 1				Session 2			
	Arrow		Gaze		Arrow		Gaze	
	Mean	SD	Mean	SD	Mean	SD	Mean	SD
Accuracy (%)								
Orienting	−0.3	2.5	0.2	1.7	−0.4	2.6	0.2	1.7
Reorienting***	−1.3	3.2	−1.0	3.9	−1.7	4.0	−0.9	3.9
Mean reaction time (ms)								
Orienting***	−6	16	−1	21	−4	16	−9	21
Reorienting***	25	21	27	19	22	16	22	20

*Note:* Asterisks indicate significant effects.

*** *p* < 0.001.

#### 3.1.1 Orienting (Valid > Neutral Cues)

We first examined the orienting process by comparing valid and neutral trials. A linear mixed model with subject as a random factor and session (first, second), cue (gaze, arrow), and trial type (valid, neutral) as fixed factors was first conducted on accuracy. No effects were significant, *F*(1, 59–266)s < 1.28, *p*s > 0.26, *f*s < 0.07, probably due to a ceiling effect.

A similar linear mixed model was also conducted on mean RT. The main effect of session was significant, *F*(1, 72) = 37.61, *p* < 0.001, *f* = 0.70, due to faster RT in Session 2 than in Session 1. The main effect of trial type was also significant, *F*(1, 171) = 10.17, *p* = 0.002, *f* = 0.23, indicating faster RT following valid cues than neutral cues. No other effects were significant, *F*(1, 153–253)s < 2.69, *p*s > 0.10, *f*s < 0.08.

#### 3.1.2 Reorienting (Invalid > Valid Cues)

We then examined the reorienting process by comparing valid and invalid trials. A linear mixed model with subject as a random factor and session (first, second), cue (gaze, arrow), and validity (valid, invalid) as fixed factors was first conducted on accuracy. The main effect of trial type was significant, *F*(1, 154) = 10.35, *p* = 0.002, *f* = 0.24, due to lower accuracy on invalid than valid trials. No other effects were significant, *F*(1, 88–254)s < 2.97, *p*s > 0.086, *f*s < 0.09.

A similar linear mixed model was then conducted on mean RT. We found a significant main effect of session, *F*(1, 76) = 44.76, *p* < 0.001, *f* = 0.75, due to faster RT in Session 2 compared to Session 1. The main effect of validity was also significant, *F*(1, 182) = 228.72, *p* < 0.001, *f* = 1.11, reflecting faster RT following valid cues than invalid cues. Furthermore, the interaction between time and cue was significant, *F*(1, 161) = 6.11, *p* = 0.015, *f* = 0.18, whereas no other significant results were found, *F*(1, 240–255)s < 0.76, *p*s > 0.39, *f* = 0.00.

To understand the interaction between time and cue, two linear mixed models were conducted for the two cues separately, with subject as a random factor and time, along with validity (added to control for its effect), as fixed factors. The *p*‐value threshold was corrected to 0.025. A significantly faster RT was observed in Session 2 compared to Session 1 for both gaze and arrow cues, *F*(1, 53–114)s > 11.41, *p*s < 0.001, *f*s > 0.44. In addition, two linear mixed models were conducted for the two sessions separately, with subject as a random factor and cue, along with validity (added to control for its effect), as fixed factors. The *p*‐value threshold was also corrected to 0.025. The results revealed significantly faster RTs on arrow trials than gaze trials in Session 1, *F*(1, 56) = 9.76, *p* = 0.003, *f* = 0.39. But faster RTs on gaze trials than arrow trials in Session 2, *F*(1, 61) = 12.57, *p* < 0.001, *f* = 0.43.

### 3.2 fNIRS Results

**FIGURE 4:**
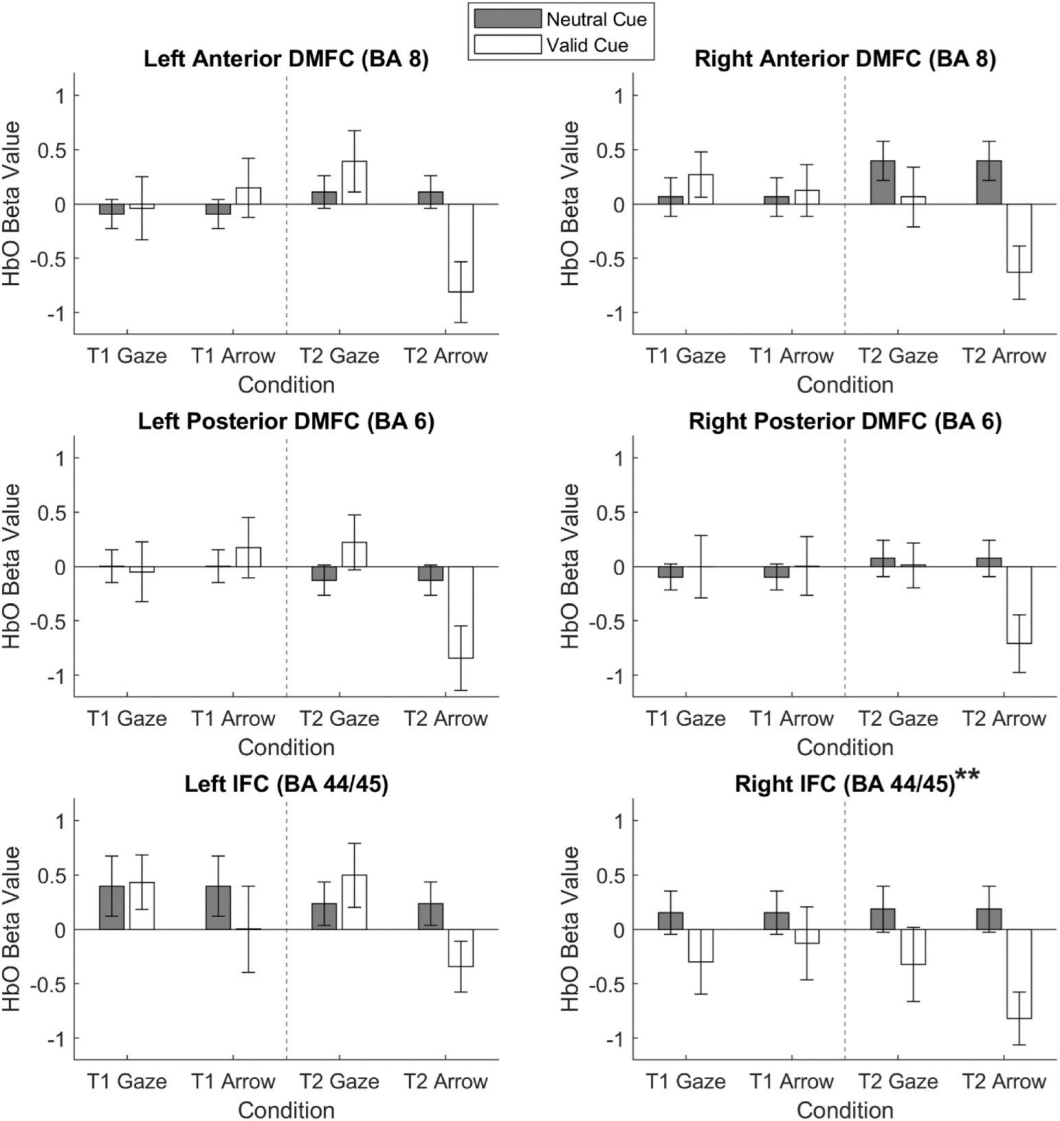
Orienting‐related (valid > neutral cues) changes in oxyhemoglobin concentration (HbO) across frontal cortex regions of interest (ROIs) on the cueing tasks. BA, Brodmann area; DMFC, dorsomedial frontal cortex; IFC, inferior frontal cortex; T1, Time 1; T2, Time 2. Error bars indicate one standard error ± the mean. Asterisks indicate regions that showed significant orienting processes after Bonferroni correction. **p < 0.0083.

**FIGURE 5:**
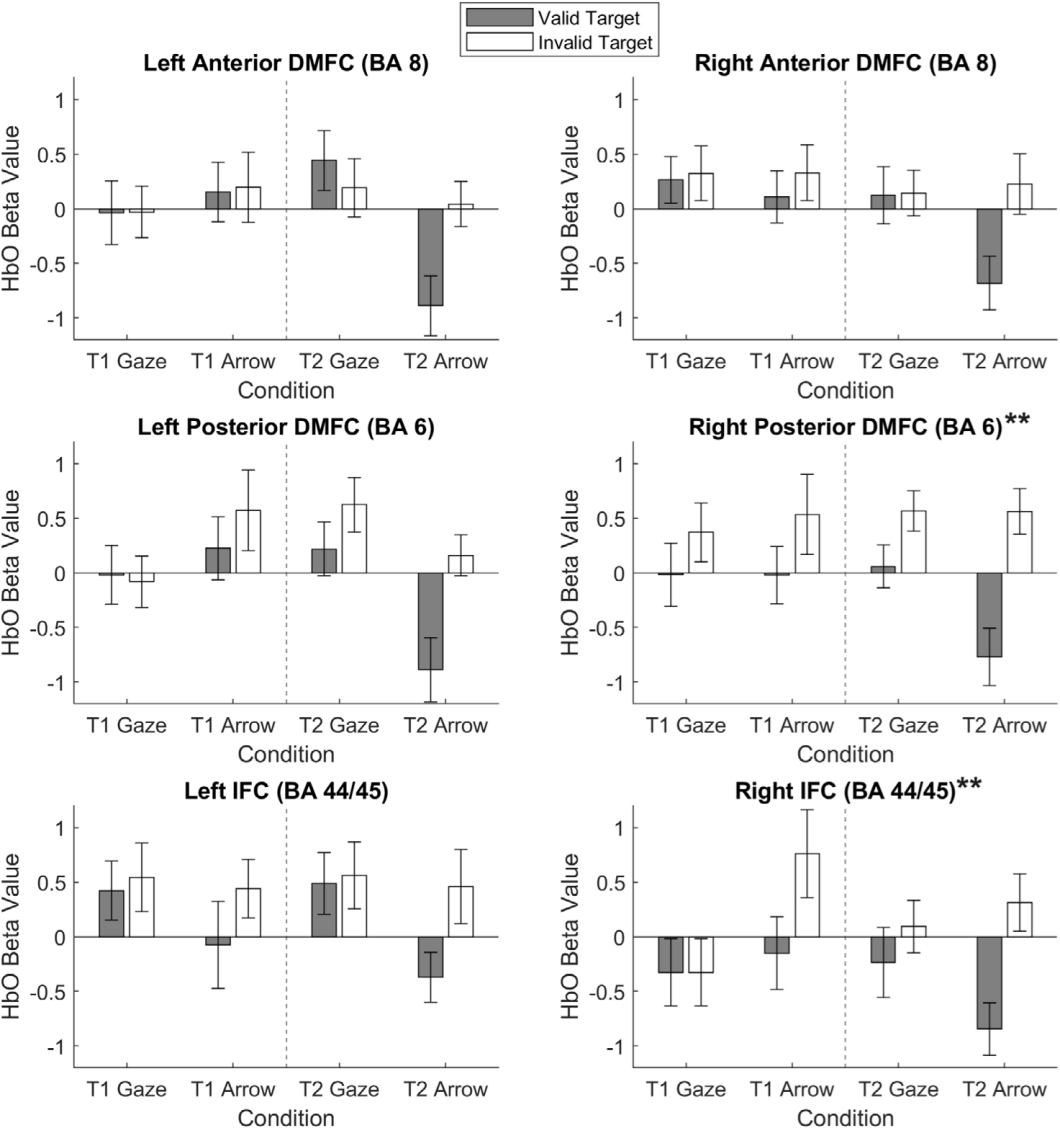
Reorienting‐related (targets following invalid > valid cues) changes in oxyhemoglobin concentration (HbO) across prefrontal cortex regions of interest on the cueing tasks. BA, Brodmann area; DMFC, dorsomedial frontal cortex; IFC, inferior frontal cortex; T1, Time 1; T2, Time 2. Error bars indicate one standard error ± the mean. Asterisks indicate regions that showed significant reorienting processes after Bonferroni correction. **p < 0.0083.

We then examined the frontal cortex processing underlying the orienting and reorienting processes. The beta values representing the changes in HbO across the six frontal cortex ROIs during the orienting and reorienting processes are shown in Figures 4 and 5, respectively. The descriptive statistics of these metrics are given in Table 2.

**TABLE 2:** Beta values of the changes in oxyhemoglobin concentration corresponding to the orienting (valid > neutral cues) and reorienting (targets following invalid > valid cues) responses.

Process	Session 1				Session 2			
	Arrow		Gaze		Arrow		Gaze	
	Mean	SD	Mean	SD	Mean	SD	Mean	SD
Left anterior dorsomedial frontal cortex (BA 8)								
Orienting	0.24	1.87	0.05	2.30	−0.92	1.85	0.28	2.05
Reorienting	0.04	3.36	0.01	2.58	0.93	2.34	−0.25	2.53
Left posterior dorsomedial frontal cortex (BA 6)								
Orienting	0.17	2.12	−0.05	1.94	−0.71	1.81	0.35	1.92
Reorienting	0.34	3.86	−0.06	2.72	1.05	2.43	0.40	2.17
Left inferior frontal cortex (BA 44/45)								
Orienting	−0.39	3.49	0.03	2.25	−0.58	1.85	0.26	2.17
Reorienting	0.51	3.09	0.12	2.45	0.83	2.73	0.07	2.75
Right anterior dorsomedial frontal cortex (BA 8)								
Orienting	0.06	2.06	0.21	1.56	−1.03	1.85	−0.33	2.22
Reorienting	0.22	2.31	0.06	2.11	0.91	2.44	0.02	1.85
Right posterior dorsomedial frontal cortex (BA 6)								
Orienting	0.10	1.63	0.10	1.90	−0.78	2.10	−0.06	1.52
Reorienting**	0.55	3.45	0.39	2.44	1.33	2.18	0.51	1.54
Right inferior frontal cortex (BA 44/45)								
Orienting**	−0.28	2.54	−0.45	2.35	−1.00	1.79	−0.51	2.54
Reorienting**	0.91	3.70	0.83	3.73	1.16	2.75	0.33	2.45

*Note:* The canonical hemodynamic response function was convolved with the onset of valid and neutral cues for orienting and with the onset of targets following invalid and valid cues for reorienting. Correlation‐Based Signal Improvement was applied. Asterisks indicate results that survived Bonferroni correction.

** *p* < 0.0083.

#### 3.2.1 Orienting (Valid > Neutral Cues)

The orienting process was examined by comparing neural activation in response to valid and neutral cues. Linear mixed models with subject as a random factor and session (first, second), cue (gaze, arrow), and trial type (valid, neutral) as fixed factors were conducted on the HbO beta values for the six ROIs. At the corrected *p*‐value threshold of 0.0083, significant results were yielded for one ROI only. For the right inferior frontal cortex (BA 44/45), the main effect of trial type was significant, *F*(1, 77) = 7.37, *p* = 0.0082, *f* = 0.28, due to lower activation on valid trials compared to neutral trials. No other effects were significant, *F*(1, 104–220)s < 0.96, *p*s > 0.33, *f*s = 0.00.

#### 3.2.2 Reorienting (Invalid > Valid Cues)

We then investigated the reorienting process by comparing neural activation in response to targets following invalid vs. valid cues. Similarly, linear mixed models with valid and invalid trials as the two trial types were conducted on the HbO beta values. At the corrected *p*‐value threshold of 0.0083, significant results were found for three of the six ROIs.

For the left posterior dorsomedial frontal cortex (BA 6), the interaction between time and cue was significant, *F*(1, 205) = 9.79, *p* = 0.002, *f* = 0.21, whereas no other effects were significant, *F*(1, 85–262)s < 3.60, *p*s > 0.061, *f*s < 0.17. Follow‐up linear mixed models were conducted separately for the two cue types, with subject as a random factor and time, along with validity (added to control for its effect), as fixed factors. The *p*‐value threshold was corrected to 0.0042 (0.0083/2). No time effects were significant, *F*(1, 48–100)s < 7.80, *p*s > 0.006, *f*s < 0.26. In addition, two linear mixed models were conducted separately for the two sessions, with subject as a random factor and cue, along with validity (added to control for its effect), as fixed factors. At the corrected *p*‐value threshold of 0.0042, a significant cue effect was found for Session 2, *F*(1, 102) = 9.07, *p* = 0.003, *f* = 0.28, but not for Session 1, *F*(1, 109) = 2.41, *p* = 0.12, *f* = 0.11. In Session 2, the change in HbO was greater on gaze trials compared to arrow trials.

To better understand this difference in left posterior dorsomedial frontal activity following gaze and arrow cues in Session 2, we examined whether the activity averaged across valid and invalid trials was above baseline (zero) or greater than that following neutral cues. This was done using one‐sample *t*‐tests (one‐tailed). The results indicated that only the social cue elicited significant activation in this region, both compared to baseline, *t*(32) = 2.20, *p* = 0.018, *d* = 0.38, and neutral cues, *t*(32) = 1.97, *p* = 0.029, *d* = 0.34. In contrast, the nonsocial cues did not elicit significant activation against baseline or neutral cues, *t*(32) = 0.00, *p* = 1.00, *d* = 0.00.

For the right posterior dorsomedial frontal cortex (BA 6), the main effect of trial type was significant, *F*(1, 86) = 10.04, *p* = 0.002, *f* = 0.32, due to a larger increase in HbO on invalid trials compared to valid trials. Similarly, for the right inferior frontal cortex (BA 44/45), the main effect of trial type was significant, *F*(1, 72) = 7.80, *p* = 0.007, *f* = 0.30, owing to a greater increase in HbO on invalid trials compared to valid trials.

## 4 Discussion

The aim of this study was to compare the mechanisms of social and nonsocial attention by examining changes over time. A cueing paradigm with spatially nonpredictive central gaze, arrow, and neutral cues, combined with fNIRS, was used to compare changes in gaze and arrow cueing effects and underlying frontal cortex processing over time. Behavioral results revealed improved RT in Session 2 compared to Session 1. They also showed a shift in the advantage of RT facilitation from the gaze cue to the arrow cue between the first and second sessions. Across cue types, neither the orienting (valid vs. neutral cues) nor reorienting (invalid vs. valid cues) significantly changed over time. In addition, fNIRS showed reduced activation in the right inferior frontal cortex during orienting and increased activation in the right posterior dorsomedial and right inferior frontal cortex during reorienting. Parallel to the behavioral finding, the gaze cue elicited greater activation in the left posterior dorsomedial frontal cortex compared to the arrow cue in Session 2.

### 4.1 The Orienting Response (Valid Versus Neutral Cues)

We found a significant orienting response that was comparable between gaze and arrow cues. Although neither cue reliably predicted the target location, participants still showed an RT facilitation effect. This finding is consistent with the automatic processing of the gaze and arrow (Friesen and Kingstone 1998; Tipples 2002), although both automatic and effortful processing likely took place (Dalmaso et al. 2020). Because there were no significant differences between cue types, social and overlearned nonsocial cues appear to elicit comparable orienting responses. The current result does not closely align with the result of Joseph et al. (2015), which failed to detect a significant orienting response following a gaze or arrow cue. The present study had a sample size of 39, adopted a mixed design, and employed schematic gaze stimuli, whereas Joseph et al. (2015) had a sample size of 20, used an event‐related design, and employed real gaze stimuli. While real and schematic faces can produce similar gaze‐cueing effects (Dalmaso et al. 2025), further studies are needed to determine whether these sample size, task design, and stimulus differences contribute to the discrepant findings.

Our fNIRS results indicated significantly reduced activation in the right inferior frontal cortex while orienting to the target location. This result is different from previous fMRI findings, which suggest a lack of significant frontal cortex involvement during the orienting process triggered by a gaze or arrow cue (Joseph et al. 2015). As mentioned above, differences in sample size, task, and stimulus characteristics may contribute to this discrepancy. The right inferior frontal cortex is a key hub in the ventral attention network (Corbetta and Shulman 2002; Vossel et al. 2014). Because our behavioral results suggest that exogenous orienting was present, the reduced activation cannot be attributed to lowered exogenous attention following the valid cue. There is substantial evidence that the right inferior frontal cortex plays a role in attentional switching, or the process by which the focus of attention is moved from one locus to another (Dove et al. 2000; Hampshire and Owen 2006; Hampshire et al. 2010). Because a specific direction was cued by the valid cue but not the neutral cue, lowering the demand to switch attention, the reduced activation in the right inferior frontal cortex might reflect lowered control demand on valid cue trials.

### 4.2 The Reorienting Response (Invalid Versus Valid Cues)

Participants showed a reorienting response, as their RT was significantly slower when the target appeared at the unattended spot compared to when the target was presented at the attended location. This observation aligns well with previous results (Greene et al. 2011; Hietanen et al. 2006; Joseph et al. 2015). We failed to detect a significant interaction between cue type and validity, which is consistent with the results of Joseph et al. (2015) but not with those of two other studies (Greene et al. 2011; Hietanen et al. 2006). Greene et al. (2011) reported a greater reorienting response following an arrow cue compared to a gaze cue, whereas Hietanen et al. (2006) detected a greater reorienting response following a gaze cue compared to an arrow cue. In the present study, the interaction between cue type and validity did not significantly change over sessions. Therefore, time does not appear to play a moderating role in the reorienting response following gaze and arrow cues.

Across cue types, there was activation in the right posterior dorsomedial frontal cortex and right inferior frontal cortex while reorienting attention from the attended to unattended location. This observation is partially consistent with those of Joseph et al. (2015), who found reorienting‐related activation in the dorsomedial frontal cortex and other cortical regions only for the gaze cue. To estimate the hemodynamic response, we convolved the canonical HRF with the target onset, the time at which valid and invalid trials started to differentiate from each other. It is unclear whether Joseph et al. (2015) convolved the canonical HRF with the cue onset, an approach adopted by other fMRI studies that compared directional and nondirectional gaze and arrow cues (Greene et al. 2011; Hietanen et al. 2006). Therefore, this methodological difference might contribute to the divergent findings.

The reorienting effect primarily reflects exogenous attention, as attention was drawn to the target appearing at an unexpected location in space. Increasing evidence suggests that reorienting is mediated by both the ventral attention network, which includes the inferior frontal cortex mainly in the right hemisphere, and the dorsal attention network, which involves the dorsomedial frontal cortex (Corbetta et al. 2008; Vossel et al. 2014). Our findings are consistent with this emerging line of understanding while contributing to the literature that activation associated with the reorienting process is robust across sessions.

### 4.3 The Differential Alerting Effects (Gaze Versus Arrow Cues)

While time did not significantly moderate the orienting and reorienting responses following cues, RT was significantly faster following arrow cues compared to gaze cues in Session 1, whereas RT was significantly faster following gaze cues compared to arrow cues in Session 2. This result suggests that participants gradually utilized the gaze cue more efficiently than the arrow cue to predict the occurrence of the target. We used gaze and arrow cues matched in visual complexity, and a double dissociation was observed. Therefore, it is implausible that the present finding simply reflects the allocation of more attention to one of the two cue stimuli. While schematic gaze cues (i.e., black pupils on white sclera) elicit similar behavior to real gaze cues even in very young infants (Michel et al. 2017), in the context of a spatial task, this effect may not be immediately apparent. The initial advantage of arrow cues may stem from the direct, learned association between an arrow and direction (cf. the arrow flanker effect). However, due to the evolutionary or social significance of gaze, the cues may gradually gain salience (Emery 2000; Stephenson et al. 2021).

Our results shed light on the findings of Hietanen et al. (2006), who reported significantly faster overall RT following an arrow cue than a gaze cue. Like the present study, schematic line draws of faces and a mixed design implementing separate blocks of directional or nondirectional gaze or arrow cues were employed. We similarly found significantly faster RT in arrow trials than gaze trials in Session 1. Hence, time plays a role in explaining the difference in the alerting response following gaze and arrow cues. Various mechanisms, such as gaze cues being more facilitative of incidental learning (e.g., regarding the cue‐target interval; Ishikawa and Yoshioka 2025) and more resistant to habituation (Liu et al. 2021) than arrows, may contribute to this time effect. Nevertheless, further research is needed to explore these possible underlying mechanisms.

The differential alerting effect was accompanied by greater activation in the left posterior dorsomedial frontal cortex following the gaze cue compared to the arrow cue in the second session. This brain region, which includes the supplementary eye field, is known to be associated with the perception of gaze (Wicker et al. 1998), the supervisory control of eye movements (Stuphorn and Schall 2002), and visually guided goal‐directed motor behavior (Nakayama et al. 2022). Additionally, the dorsomedial frontal cortex is an executive hub for selecting stimuli and responses (Asanowicz et al. 2021) and for initiating and sustaining a motor response (Stuss 2011). Therefore, the imaging results may suggest that individuals gradually develop improved initiation, selection, monitoring, and control of oculomotor behavior in response to gaze cues over time.

### 4.4 Implications and Limitations

Our findings of differential changes in gaze and arrow alerting effects over time have important implications for a more nuanced understanding of social attention across various populations, particularly in autism (Yeung 2022), which is characterized by social communication deficits. Altered processing of gaze cues has been observed in some studies but not consistently in autistic individuals (Kuhn et al. 2010; Zhao et al. 2017). Investigating whether, as we found, there is an increase in the alerting effects of gaze cues along with increased engagement of the left posterior dorsomedial frontal cortex over time in the autistic population will provide insights into the dynamic aspects of social attention among autistic individuals. Additionally, this study offers preliminary fNIRS data that can facilitate subsequent applications of fNIRS, which is an increasingly popular method for exploring brain functions in autism (Zhang and Roeyers 2019), to better understand the neural mechanisms underlying the temporal changes in social attention in these populations.

Notwithstanding these contributions, the present study has some limitations. First, our fNIRS measurements were confined to the frontal cortex; other regions involved in the dorsal and ventral attention networks, such as the intraparietal sulcus and temporoparietal junction (Corbetta et al. 2008; Vossel et al. 2014), were not assessed. Therefore, fMRI studies are needed to provide a more comprehensive picture of the functional changes within these attention networks underlying the differential changes in gaze and arrow alerting effects over time. Second, we employed only a single cueing paradigm, and our sample consisted solely of young healthy adults. Consequently, further research is necessary to determine whether these findings generalize to other experimental paradigms and populations.

### 4.5 Conclusion

In summary, the present findings suggest that the orienting and reorienting processes triggered by spatially nonpredictive central gaze and arrow cues are comparable and do not systematically change over time. However, gaze cues gradually elicit a more efficient alerting process than arrows, which is accompanied by increased engagement of the left posterior dorsomedial frontal cortex, a region implicated in the instigation and higher‐order control of oculomotor movements. Taken together, insofar as changes over time are concerned, gaze and arrow follow partially distinct attentional and neural mechanisms.

Over the years, various individual and social factors (Dalmaso et al. 2020; McKay et al. 2022), as well as task characteristics (McKay et al. 2021), have been identified that modulate the gaze cueing effect. Therefore, future neuroimaging studies that compare stimulus and task features and identify factors influencing the social cueing effects would facilitate a more comprehensive understanding of the neural mechanisms of social attention.

## Author Contributions

M.K.Y. contributed to the conceptualization, methodology, data curation, investigation, formal analysis, and writing of the original draft. Y.M.Y.H. contributed to the conceptualization, methodology, investigation, resources, and the review and editing of the manuscript.

## Conflicts of Interest

The authors declare no conflicts of interest.

## Data Availability

The dataset and data processing scripts that form the basis of the results are available on OSF (https://osf.io/p7cx4).

## Endnotes

^1^
There were three block types, namely gaze, arrow, and neutral cue blocks. To compare the level of sustained attention among block types, the canonical hemodynamic response function was convolved with the block duration using a boxcar function, and a general linear model analysis was conducted. Linear mixed model analyses with subject as a random factor and block type (gaze, arrow, neutral cue) as a fixed factor revealed no significant effects of block type on activation across ROIs, *p*s > 0.05.
